# Immune Responses to AAV-Vectors, the Glybera Example from Bench to Bedside

**DOI:** 10.3389/fimmu.2014.00082

**Published:** 2014-03-03

**Authors:** Valerie Ferreira, Harald Petry, Florence Salmon

**Affiliations:** ^1^Research and Development, uniQure B.V., Amsterdam, Netherlands

**Keywords:** adeno-associated viral vectors, gene therapy, alipogene tiparvovec, immune responses, clinical safety, clinical efficacy

## Abstract

Alipogene tiparvovec (Glybera^®^) is an adeno-associated virus serotype 1 (AAV1)-based gene therapy that has been developed for the treatment of patients with lipoprotein lipase (LPL) deficiency. Alipogene tiparvovec contains the human LPL naturally occurring gene variant LPL^S447X^ in a non-replicating viral vector based on AAV1. Such virus-derived vectors administered to humans elicit immune responses against the viral capsid protein and immune responses, especially cellular, mounted against the protein expressed from the administered gene have been linked to attenuated transgene expression and loss of efficacy. Therefore, a potential concern about the use of AAV-based vectors for gene therapy is that they may induce humoral and cellular immune responses in the recipient that may impact on efficacy and safety. In this paper, we review the current understanding of immune responses against AAV-based vectors and their impact on clinical efficacy and safety. In particular, the immunogenicity findings from the clinical development of alipogene tiparvovec up to licensing in Europe will be discussed demonstrating that systemic and local immune responses induced by intra-muscular injection of alipogene tiparvovec have no deleterious effects on clinical efficacy and safety. These findings show that muscle-directed AAV-based gene therapy remains a promising approach for the treatment of human diseases.

## Introduction

Adeno-associated virus (AAV) is a naturally occurring virus that is known to infect humans and other primates. It is expected to interact at multiple levels with the innate and adaptive immune system and elicit immune responses when injected in man. AAV-based vectors are nowadays often chosen for the development of new, promising gene therapy approaches because of several interesting features such as their inability to self-replicate. However, a potential concern about the use of such virus-derived vectors is the potential to induce humoral and cellular immune responses in the recipient that may impact on efficacy and safety. In this report, we review the current understanding of immune responses against AAV-based vectors and their impact on clinical efficacy and safety using alipogene tiparvovec (Glybera^®^) as an example. Glybera^®^ has received marketing authorization under exceptional circumstances in Europe in 2012. Alipogene tiparvovec is an AAV-based gene therapy vector that has been developed for the treatment of patients with lipoprotein lipase deficiency (LPLD). It contains the gene of the naturally occurring gain-of-function variant LPL^S447X^ of the human lipoprotein lipase (LPL) in a non-replicating viral vector based on adeno-associated virus serotype 1 (AAV1). The immunogenicity findings from the clinical development of alipogene tiparvovec will be discussed, demonstrating that systemic and local immune responses induced by intra-muscular injection of alipogene tiparvovec have not shown deleterious effects on clinical efficacy and safety. Alipogene tiparvovec is an example that muscle-directed AAV-based gene therapy is a promising approach for the treatment of human diseases.

### Particular features of AAV and AAV-vectors

Wild-type AAV is not associated with any known disease or pathology in humans (1, 2). In addition, the virus is naturally replication-defective and requires a helper virus such as adenovirus to replicate (3). Wild-type AAV also has been shown *in vitro* to have the ability to stably integrate into the host cell genome at a specific site (designated AAVS1) in the human chromosome 19 with minimal risk for random incorporations into the genome. For these reasons, AAV has attracted considerable interest because of its potential as a gene therapy vector. The use of AAV as gene therapy vectors has required the elimination of the rep gene from the vector, since it is coding for the protein that is involved in replication of the viral DNA and site-specific integration. In the vector genome, the rep and cap genes are replaced by the transgene, in the case of alipogene tiparvovec the gene for LPL, together with a promoter that is necessary to drive transcription. This cassette is flanked by inverted terminal repeats (ITRs) that are necessary for the formation of so-called concatemers in the nucleus after the single-stranded vector DNA is converted by host cell DNA polymerase complexes into double-stranded DNA. These episomal concatemers remain intact in the nucleus of non-dividing host cells. Hence, transferred genomes tend to persist inside the cells mainly in this episomal, non-integrated form (4, 5). The generation of AAV-vectors currently used for gene therapy in humans has strongly reduced the risk of insertional mutagenesis (6–8). As a result, AAV-vectors are among the simplest gene therapy vectors, containing only the transgene expression cassette flanked by two non-coding viral ITRs, and enclosed in a capsid composed of three structural proteins, VP1, 2, and 3 (9). Alipogene tiparvovec indeed is such an AAV-vector and contains the transgene coding for LPL^S447X^.

Another important feature of AAV and also of AAV-based vectors is their very low immunogenic potential. Immune responses against AAV in general seem restricted and mainly consist in the generation of neutralizing antibodies, while well-defined cytotoxic responses seem minimal (10). This feature, along with the ability to infect quiescent cells, is another important advantage for AAV for their use as vectors for human gene therapy. Presumably several features of AAV contribute to this low immunogenicity, including the simplicity of AAV-vectors and their low efficiency in transducing professional antigen presenting cells such as macrophages or dendritic cells, and their lacking capacity to express viral proteins (11, 12).

### Non-clinical investigations on the immunogenicity of AAV-vectors

A large number of studies in various animal species have demonstrated the potential of AAV-vectors as a therapeutic platform for gene delivery (13–22). However, the AAV capsid protein as well as the transgene product can interact at multiple levels with the innate and adaptive immune system. Consistent with current concepts in immunology, the immune response can vary substantially depending upon the tissue which is targeted, with outcomes ranging from almost unresponsiveness (gene transfer in the eye or in the brain) to responsiveness (gene transfer in the muscle, liver, or lung). Humoral immune responses to AAV capsid proteins were reported in all animal studies in which AAV-vectors were used to target muscle or liver. While cellular and humoral immune responses to AAV were reported to be modest in intensity in mouse models (23–25), cytotoxic T-cell responses to AAV-vector and transgene product in muscle of large animal models have been recently reported, which emphasizes the importance of appropriate animal models to address safety and efficacy of the approach and predict clinical outcomes (26).

### Clinical studies with AAV-gene therapy vectors in humans

Over the last two decades, numerous clinical studies were performed using AAV to deliver therapeutic genes to different organs and tissues including muscle, liver, and the CNS. Those studies included hundreds of patients and indicate an excellent safety record of AAV-vectors for gene therapy in humans. The different safety aspects of AAV for the use in humans have recently been summarized elsewhere [see for review Ref. (23, 26, 27)].

Immune responses have been assessed in clinical trials by measuring systemic and local cytotoxic reactions as well as (neutralizing) antibodies against AAV and/or the expressed therapeutic protein (24, 25, 28–37). Results from these measurements in these clinical studies indicate that the immune responses measured could impact on the efficacy of the product rather than the overall safety profile. The immunogenicity data reported so far show that immune responses against AAV capsid proteins can vary widely and amongst others are influenced by the target organ, route of delivery, and dosing schedule.

The eye and central nervous system are known to be immune-privileged compartments of the body due to adaptations that limit immune responses. Delivery of AAV-vectors directly into the brain has been tested in a number of studies (31, 38–40). Similarly, subretinal vector delivery has been performed in a number of clinical studies (28, 33). In all these studies, AAV-vector administration was associated with little or no detectable immune response to the capsid or the transgene protein in serum and peripheral blood mononuclear cell (PBMC). In contrast, humoral immune responses to AAV capsid proteins were reported in all trials targeting AAV-based vectors to muscle or liver.

Cellular immune responses against the AAV-vector have been found in only some of the clinical trials performed. The first observation of a cellular immune response induced by AAV-gene therapy to our knowledge was in patients with hemophilia B who were treated with an AAV-vector to deliver human coagulation factor IX (24, 27, 41, 42). In this study, a cell-mediated immune response to AAV2 capsid was reported, which was measured in parallel with a loss of transgene expression. In a more recent clinical study in patients with hemophilia B, using the capsid of AAV8 to deliver FIX to the liver, similar reactions were observed in some of the patients treated with the highest dose (37). Whereas both studies in patients with hemophilia B indicate a direct correlation between the induction of a CD8 T-cell response toward the AAV capsid proteins and a loss of transgene expression, this seems not to be the case after intra-muscular administration of an AAV-vector. In a clinical study in patients with α-1 antitrypsin (AAT) deficiency in which the gene for AAT was delivered by an AAV1 capsid, cellular immune responses were found against the capsid proteins from day 14 in all subjects. However, the influence of those T-cells is not clear since the expression of the transgene was sustained at sub-therapeutic levels in all subjects. These data suggest that the cellular immune responses to the AAV capsid did not eliminate the transduced cells (25). Similarly, systemic and local cellular immune responses induced by intra-muscular injection of alipogene tiparvovec did not appear to have an impact on safety and did not prevent clinical efficacy (43). However, cellular host immune responses to both AAV capsid and transgene products have been shown in the context of muscular dystrophy (26).

## Clinical Studies with Alipogene Tiparvovec

**Figure 1:**
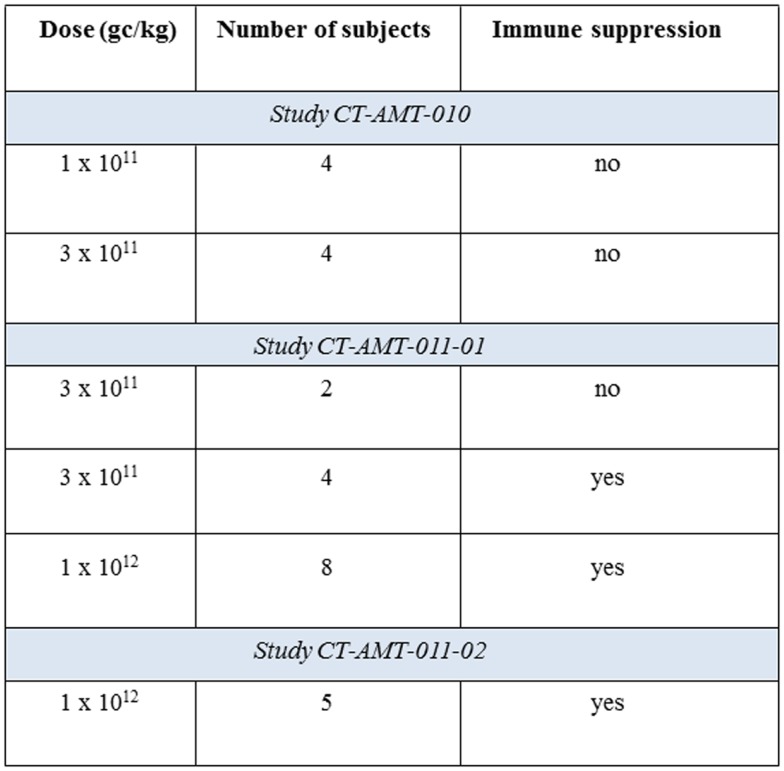
**Summary of the clinical studies with alipogene tiparvovec**.

Alipogene tiparvovec (called AAV1–LPL^S447X^ in the early phases of clinical development) is an AAV1 vector expressing a naturally occurring variant of the LPL transgene, LPL^S447X^, associated with improved lipid profile and carried by approximately 20% of the general population (44). Building on successful proof-of-concept studies in animal models (45, 46), three interventional clinical studies have been conducted with alipogene tiparvovec in patients with LPL-deficiency (CT-AMT-010, CT-AMT-011-01, CT-AMT-011-02) (Figure 1). In all studies, alipogene tiparvovec was administered via multiple direct intra-muscular injections into the lower extremities in the subjects with LPL-deficiency. Alipogene tiparvovec was administered with a 22 gage needle as multiple injections of 0.5 ml volume (maximum) with a distance of 2.5–3 cm between each site. The total number of injections was divided equally between the vastus lateralis and vastus medialis of both the left and right musculus femoralis. The calf muscles were also injected if the number of injections exceeded 40 injection sites. Both the muscle and major blood vessels were identified prior to injection using ultrasound to ensure intra-muscular administration, and to avoid intra-vascular injection. Two injection sites were labeled with permanent skin tattoos so that injection sites could be identified for subsequent biopsy. The total dose delivered to subjects was calculated based upon the subject’s body weight and his/her allocation to a specific dosing cohort.

### Clinical development program

#### Study CT-AMT-010

Eight patients with LPL-deficiency were first monitored for several months, and then treated once with multiple intra-muscular injections of AAV1–LPL^S447X^ (predecessor of alipogene tiparvovec containing the same construct but manufactured in another cell system). The patients did not receive immunosuppressants pre- or post-exposure to AAV1–LPL^S447X^, and were followed-up for up to 18 months after administration in the active phase of the study.

#### Study CT-AMT-011-01

After an observation period of a few months, 14 patients with LPL-deficiency were treated with alipogene tiparvovec. Twelve of these patients received immunosuppressants. The immunosuppressant regimen consisted of cyclosporine A at a dose of 3 mg/kg/day and mycophenolate mofetil at a dose of 2 g/day and was maintained until 12 weeks after administration of alipogene tiparvovec. The patients were followed-up for 5 years after administration.

#### Study CT-AMT-011-02

After a run-in phase of a few months, five patients with LPL-deficiency were treated with alipogene tiparvovec. All patients have received immunosuppressants, starting shortly before exposure to alipogene tiparvovec. The immunosuppressant regimen consisted of 3 mg/kg/day cyclosporine A, 2 g/day mycophenolate mofetil, and a bolus injection of methylprednisolone was given 30 min prior to alipogene tiparvovec administration. The immunosuppressant regimen with cyclosporine A and mycophenolate mofetil was maintained until 12 weeks after exposure. The patients were followed-up for a year after administration in the active phase of the study.

**Figure 2:**
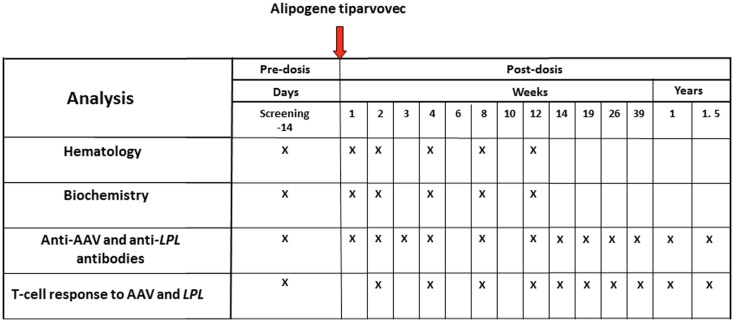
**Clinical analyses schedule of the studies with alipogene tiparvovec until 1.5 years after drug delivery**.

A summary of the clinical analyses schedule with alipogene tiparvovec is given in Figure 2. The follow-up scheme included routine hematology and biochemistry up to 3 months, immunology monitoring up to 1.5 years, and a biopsy of the injected muscle. No hematology visits were planned after week 12. Antibodies as well as cellular responses against AAV1 and LPL were monitored in the long-term follow-up at 2, 3, 4, and 5 years after administration of alipogene tiparvovec. Blood samples were obtained from all subjects enrolled in the clinical trials pre- and up to 5-year post-administration of alipogene tiparvovec, and analyzed for the presence of total antibodies against AAV1 capsid proteins and LPL by ELISA-based assay. In addition, all blood samples were tested for presence of T-cells specific for AAV with an enzyme-linked immunospot (ELISpot) assay. Of note, patients with pre-existing total antibodies against AAV1 were included in the clinical trials. Biopsy specimens of the injected muscle as well as specimens from the non-injected muscle (control) were taken between 10 and 52 weeks after injection for immunohistochemical studies. The specimens were analyzed for the presence and the nature of any cellular infiltrates. In addition, blood samples were tested at regular intervals for inflammation markers such as C-reactive protein (CRP) and neutrophil counts, as well as for parameters reflecting local (inflammatory) muscle damage such as creatine phosphokinase (CPK).

### Immunological measurements in the clinical program of alipogene tiparvovec

#### Antibodies against AAV1 capsid proteins

Humoral (total antibodies) responses against AAV1 capsid proteins were measured with an ELISA assay. Briefly, AAV1 capsid proteins were fixed to polystyrene ELISA plates and incubated with the serum samples to be tested. Bound antibodies were detected by a subsequent incubation with conjugated antibodies against human immunoglobulins. The ELISA did not discriminate between IgG subclass antibodies. To discriminate between samples with normal and with elevated levels of anti-AAV antibodies, a cut-off level was established using serum samples from 30 healthy volunteers. The test results of the samples to be tested were scored by comparison with those of the negative control, which was a serum sample from a healthy human control. To this end, algorithms were developed to convey the optical density results into a semi-quantitative scoring system. Based on the algorithms, samples were said to be strongly positive (++), weakly positive (+), or negative (−) for AAV1 total antibodies.

#### Antibodies against LPL

Antibody responses against LPL^S447X^ were assessed by measuring total antibody levels in pre- and post-exposure serum samples with an ELISA assay. This ELISA was similar to that described above for antibodies against AAV1 proteins, except that recombinant LPL was used to coat plates. The recombinant LPL^S447X^ was isolated from medium of stably transfected CHO cells that express LPL^S447X^. The ELISA did not discriminate between IgG subclass. A cut-off level of the assay was established in a similar way as described in the previous paragraph for anti-AAV1 antibody ELISA. Also for this ELISA, an algorithm was developed to convey the optical density results into a semi-quantitative scoring system. Based on the algorithms, samples were said to be positive (+) or negative (−) for anti-LPL antibodies.

#### Assay for AAV1-specific T-lymphocytes

In order to monitor the T-cell-mediated immune response in the patients treated with alipogene tiparvovec, an ELISpot assay was developed. This assay is based on the detection and quantification of interferon gamma (IFN-γ) secreting cells upon stimulation with AAV1 capsid antigens. To this end, PBMCs were obtained from the patients and incubated with AAV1 capsid antigens. A one color ELISpot assay was used for this purpose as previously described (23, 24) and further validated by a contract research laboratory, SeraCare Life sciences (Milford, MA, USA), according to predefined QA/QC specifications.

Two criteria are widely used to evaluate test results of ELISpot assays, which are the number of spot forming unit (SFU) per million cells upon stimulation with antigen and the increase of SFU number compared to that in medium only. Generally, samples are said to be positive for T-cells when upon stimulation with antigen they contain >50 SFU per million cells, and when that number is at least threefold higher than that of the medium control. These criteria were also used to assess T-cells against AAV1.

#### Immunohistochemical analysis

**Figure 3:**
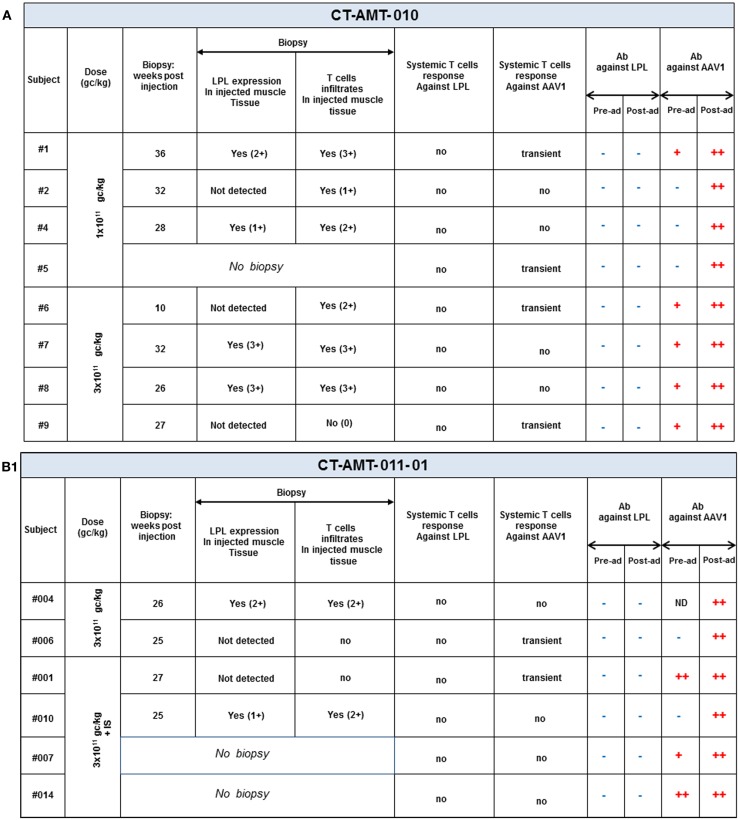

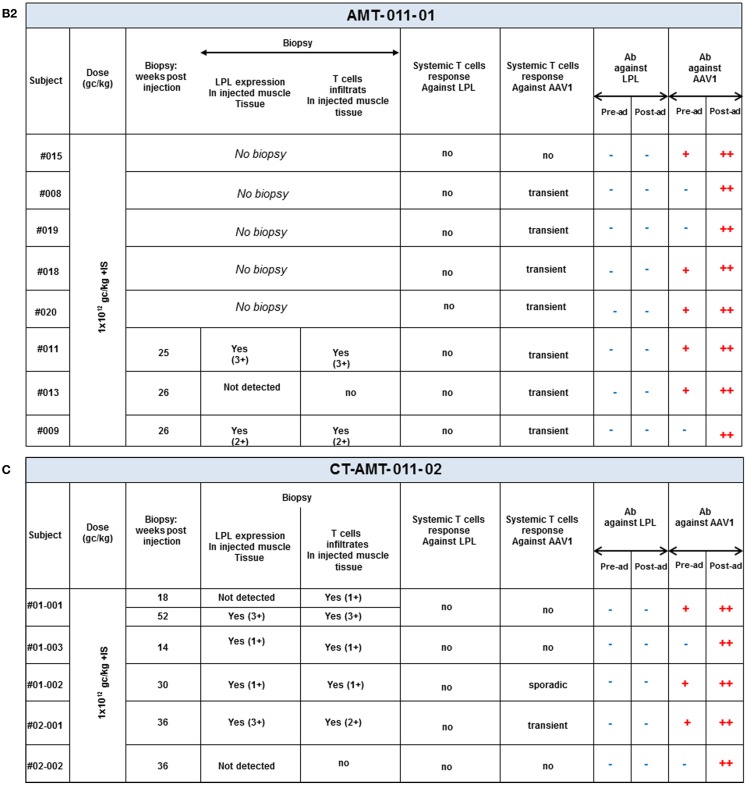
**A per-patient summary of the immunological data obtained in study CT-AMT-010 (A), CT-AMT-011-01 (B), and CT-AMT-011-02 (C)**. Biopsies: the scoring of LPL-expression in injected muscle tissue reflect the number of cells positive for lipid deposits, as identified using oil red O staining of cross-sections: 0, −, 1+, rare; 2+, moderate; 3+, high number. The scoring of T-cells infiltrates in injected muscle tissue reflect the number of infiltrates observed, as identified using staining of cross-sections with cell-specific markers: 0, −, 1+, rare; 2+, moderate; 3+, high number. It should be noted that the scores given are arbitrary, simply providing a semi-quantitative or relative means to distinguish between patients in terms of amount of inflammatory cells observed. As such, a score of 3+ represents the highest score observed in the study. Systemic T-cells responses: samples were said to be positive for T-cells when upon stimulation with antigen they contained >50 SFU per million cells, and when that number was at least threefold higher than that of the medium control. A T-cell response to the antigen was reported transient positive (transient) when at least two consecutive sampling time points were measured positive in the ELISpot assay. The patient reported “sporadic” present recurrent non-consecutive T-cell response over time. When none or only one sampling time was measured positive, the T-cell response was reported negative (−). Antibodies: the test results of the samples were scored by comparison with those of a negative control (a serum sample from a healthy human control). To this end, algorithms were developed to convey the optical density results into a semi-quantitative scoring system. Based on the algorithms, samples were said to be strongly positive (++), weakly positive (+), or negative for AAV1 antibodies and positive (+) or negative (−) for anti-LPL antibodies.

Open muscle biopsies were collected between 10 and 52 weeks after intra-muscular injection of AAV1–LPL from both an injected (tattooed) muscle (vastus lateralis) and a non-injected control muscle site (not tattooed musculus tibialis anterior). The specimens were analyzed according to routine evaluations, including muscle histology and immunohistochemical characterization of infiltrating cells when present. The biopsy procedures were performed at variable time after injection mainly dependent on the availability of the patients. The predefined criteria for the collection of tissue specimens in the protocol were 14 weeks for the first biopsy and 52 weeks for the follow-up biopsy, independently of any clinical indication. However, due to the availability of the patients, deviations from the protocol occurred and the real time of collection of the biopsy specimens is reported for each patient in Figure 3. Specimens from the injected muscle were compared to those from the contralateral non-injected muscles from each subject. The histological assessments were carried out according to routine procedures at the Department of Neuropathology, Academic Medical Centre (AMC), Amsterdam, The Netherlands, by Dr. Dirk Troost and Dr. Eleonora Aronica, both specialized in the histopathology of human muscle. Tissues’ scoring was expressed as negative to 3+ positive.

## Analysis of the Treatment-Emergent Immune Responses in the Patients Treated with Alipogene Tiparvovec

An overview of the systemic as well as local humoral and cellular immune responses observed in all patients participating in the clinical studies is shown in Figures 3A–C. It should be noted that only 19 patients gave their consent to have muscle biopsies taken, 7 patients from study CT-AMT-010, 7 from study CT-AMT-011-01, and 5 from study CT-AMT-011-02.

### Humoral immune responses upon treatment with alipogene tiparvovec

Fifteen of the 26 subject had pre-existing antibodies against AAV1. Among the 19 patients of whom a post-exposure biopsy was available, 11 had pre-existing anti-AAV antibodies. No apparent relationship was found between pre-existing AAV1 antibodies and LPL-expression after administration of alipogene tiparvovec: 7 of the 11 patients with pre-existing anti-AAV1 antibodies had LPL-expression in the biopsy versus 4 of the 7 patients with no such antibodies (Figure 3). As expected, and in line with published data observed with other AAV-vectors all subjects, whether exhibiting pre-existing antibodies or not, showed a treatment-emergent anti-AAV1 total antibody response, which became detectable at 1 or 2 weeks after the admginistration of alipogene tiparvovec. The anti-AAV1 total antibody titers measured at those early time points remained stable over the whole observation period. The responses of circulating antibodies against AAV1 observed in the clinical development program with alipogene tiparvovec are consistent with data reported for other published gene therapy trials with AAV-based vectors [among others, Ref. (25, 41, 42, 47)]. In each patient, an increase in the level of anti-AAV antibody titers was observed upon administration of alipogene tiparvovec, which was sustained overtime. There was no apparent difference in anti-AAV1 antibody response between studies and dose cohorts suggestive that the dose and/or the immunosuppressive regime did not influence anti-AAV1 total antibody formation.

None of the patients had anti-LPL antibodies prior to the administration of alipogene tiparvovec nor developed those after delivery of the product. The baseline levels of anti-LPL antibodies were below the cut-off level of detection in all patients prior to administration of alipogene tiparvovec. Post-administration levels remained below the cut-off level, demonstrating that no antibody responses were mounted against the expressed LPL protein after administration of alipogene tiparvovec even in the long-term.

### Cellular immune responses upon treatment with alipogene tiparvovec

**Figure 4:**
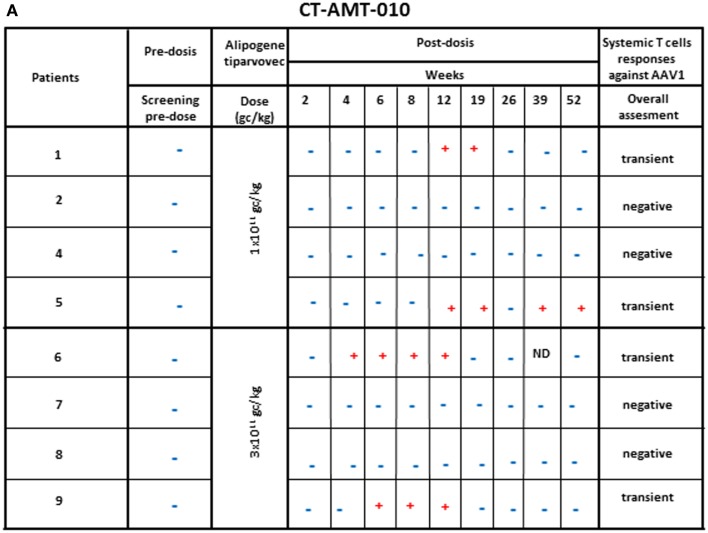

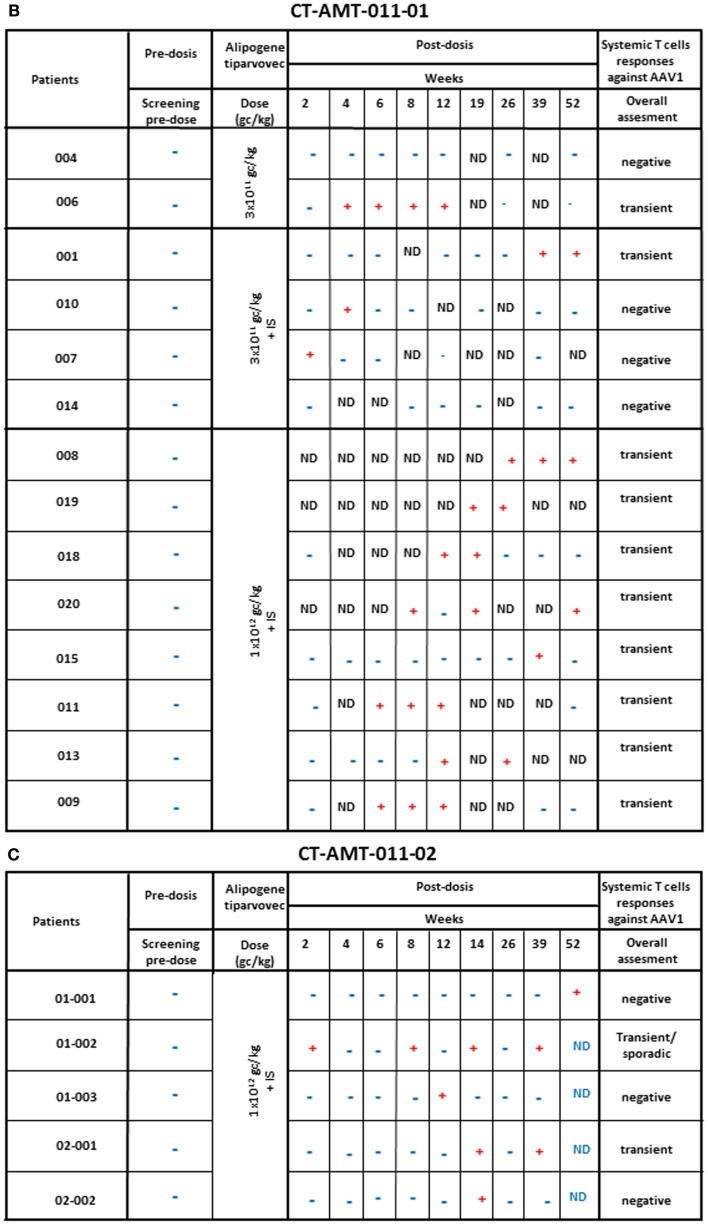
**Systemic cellular immune responses following alipogene tiparvovec administration**. The tables below provides an overview of the individual patient systemic T-cell response against AAV1 overtime for study CT-AMT-010 **(A)**, study AMT-011-01 **(B)**, and study AMT-011-02 **(C)**. We considered that a subject developed a T-cell-mediated immune response to AAV1 capsid proteins when at least two of the eight to nine sampling time points were measured positive (+) in the ELISpot assay. When only one sampling time was reported positive (+), the T-cell response was considered negative.

Enzyme-linked immunospot assays were used to assess the course of numbers of T-cells in peripheral blood that were directed against AAV1 epitopes. The results obtained across studies and per subjects are given in Figures 4A–C. In study AMT-010 (48), the intra-muscular administration without immune suppression of the AAV1 vector in humans resulted in a transient T-cell activation in four of eight subjects. In study AMT-011-01 (49), cellular immune responses against AAV1 capsid proteins were also observed despite immune suppression. On the basis of the data obtained from PBMCs of adequate quality, a moderate and non-persistent T-cell response was observed directed against the AAV1 capsid in 9 out of the 14 subjects. Therefore, the immunosuppressive regimen was further optimized in study CT-AMT-011-02 (50) by starting administration of cyclosporine and MMF administration earlier before alipogene tiparvovec administration, and by adding a bolus injection of methylprednisolone at the time of alipogene tiparvovec administration. However, in study CT-AMT-011-02, comparable cellular immune responses against AAV1 capsid proteins were observed as in study CT-AMT-011-01. Overall, transient cellular immune responses did not have clinical consequences as they were not associated with any clinical signs or symptoms such as persistent elevation of blood levels of CRP, CPK, or other inflammation markers (see Adverse Events).

Enzyme-linked immunospot assays were also used to evaluate whether there was any T-cell responses against the expressed LPL protein product. In none of the patients, such immune responses were observed upon administration of alipogene tiparvovec at any dose.

### Local T-cell responses

Biopsy specimens of the injected muscle from 19 patients were available for immunohistochemical analyses. As described previously, these specimens were assessed for the presence of inflammatory cell infiltrate by qualified histopathologists. Infiltrates of varying intensity consisting of B cells, macrophages, and mainly T-cells, were found in 15 patients whereas in the other 4, no inflammatory cell infiltrate was observed. However, as illustrated in study AMT-011-02 with subject 02-002 for which no infiltrates in the injected muscle was detected, the biopsy of some subjects was taken weeks after the peak of systemic T-cells response reached baseline, compared to a few weeks for others subjects. Therefore, the time of the biopsy should be taken into consideration when putting in parallel local and systemic T-cell responses.

As none of the patients developed a T-cell response against LPL, the relation between an immune response to the transgene and the presence of a local T-cell infiltrate was not further investigated. The presence of cytotoxic T-lymphocytes (CTL) in the inflammatory cell infiltrates was investigated in the muscle tissues injected with alipogene tiparvovec as well as in muscle biopsies from non-injected muscle. As a parameter for cellular cytotoxicity, granzyme B and Fas ligand expression by the cells was measured. CD8-positive T-cells in the infiltrates were negative for granzyme B and Fas ligand expression, which suggest that the majority of the T-lymphocytes present in the injected muscle tissue lack cytotoxic properties. Furthermore, CD4-positive T-cells observed in muscle biopsies from LPLD subjects injected with alipogene tiparvovec were further assessed for the expression of the transcription factor FoxP3, as a marker for regulatory T-cells. FoxP3-positive/CD4-positive T-cells were also found in the infiltrates suggesting that multiple mechanisms contribute to the local immune tolerance to alipogene tiparvovec administration (50).

## Immunogenicity of Alipogene Tiparvovec and Efficacy

The presence of LPL protein in the muscle biopsies and the improved clearance of post-prandial chylomicrons levels in plasma were used to measure local and systemic activity of alipogene tiparvovec and were considered as efficacy markers (49, 51). However, the use of muscle biopsies has several hindrances. At first, out of the 27 patients treated with alipogene tiparvovec, 19 patients gave their consent for a muscle biopsy once. Only one patient allowed the procedure to be done twice. Second, the results were influenced by the limited spread of the product in the muscle tissue and the variability in the procedure, as not all biopsies were consistently taken in close proximity to the actual injection site. Therefore, the circulating chylomicrons plasma levels measurement was developed in parallel to the clinical study CT-AMT-011-01 and used as a primary end point only in the study CT-AMT-011-02, as the most reliable endpoint to determine the systemic activity of the LPL enzyme.

Fifteen of the 26 patients, with registered data for presence of pre-administration anti-AAV1 antibodies, had pre-existing antibodies against AAV1. Among the 15 patients with pre-existing antibodies against AAV1, biopsies were obtained from 11 patients. Among those 11 patients, 7 had LPL-expression in the biopsy. In comparison, from the 11 patients without pre-existing antibodies, 8 muscle biopsies were obtained; and from those, 5 were stained positive for LPL-expression. This distribution was very similar across the three studies CT-AMT-010, CT-AMT-011-01, and CT-AMT-011-02. Our results strongly indicate that there was no apparent relationship between the presence of pre-existing AAV1 antibodies in LPLD patients and LPL-expression after administration of alipogene tiparvovec.

After the administration of alipogene tiparvovec in all 27 patients, the development of treatment-emergent antibodies against AAV1 capsid proteins was observed, independently of whether pre-existing antibodies were present or not. In relation with efficacy, those treatment-emergent antibody responses against the AAV1 capsid proteins upon treatment with alipogene tiparvovec, did not seem to affect expression of the transgene.

A similar conclusion as drawn for the presence and development of AAV1-specific antibodies can be drawn for the development and presence of AAV-specific T-cells after administration of alipogene tiparvovec. The percentage of patients with treatment-emergent T-cell response across studies and among the three dose groups was 50% (2/4) of the subjects treated with 1 × 10^11^ gc/kg having a positive response, 40% (4/10) of the patients treated with 3 × 10^11^ gc/kg, and 69% (9/13) of the patients treated with 1 × 10^12^ gc/kg, which suggest an AAV1-dose-dependent kinetics of T-cell response appearance (48–50). Of the 19 patients who consented to the biopsy procedure, 10 developed AAV1-specific T-cells. Of those 10 patients, 5 were tested positive for the expression of LPL in the biopsies. Of the 9 patients with no detectable T-cell response against AAV1, 7 had detectable LPL-expression in their biopsy. In relation with efficacy, those transient T-cell responses against the AAV1 capsid proteins upon treatment with alipogene tiparvovec, did not seem to directly influence the expression of the transgene.

However, as mentioned previously, the variability of the biopsy procedure and by consequence, the difficulty for quantification and comparison between patients has to be considered in the interpretation of the data. The differences in the results obtained with the biopsies of patient 01-001 (in study CT-AMT-011-02), collected at 18 and 52 weeks after administration of alipogene tiparvovec, illustrate this issue. The biopsy taken at week 18 yielded no detectable LPL-expression, whereas the biopsy taken at week 52 yielded a strong expression of LPL.

**Figure 5:**
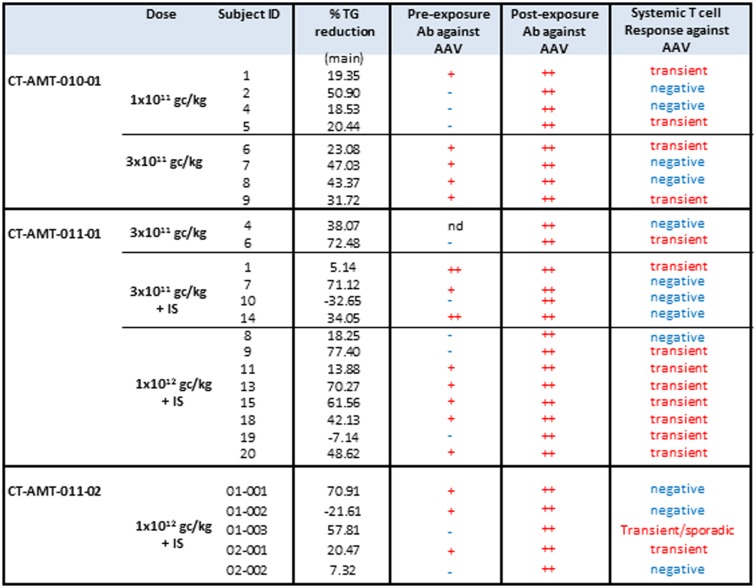
**TG responder status in relation with humoral and systemic T-cell response against AAV**. The table below provides an overview of the individual patient cellular and humoral immune responses in relation with the clinical end point (fasting) total plasma TG. A T-cell response to the antigen was reported transient positive (transient) when at least two consecutive sampling time points were measured positive in the ELISpot assay. The patient reported “sporadic” present recurrent non-consecutive T-cell response over time. When none, or only one sampling time was reported positive, the T-cell response was reported negative (−).

The administration of alipogene tiparvovec resulted in functional LPL activity levels sufficient to achieve a beneficial clinical effect in patients with LPLD. This conclusion is supported by the evidence that levels of plasma TG decreased in LPLD patients after administration of alipogene tiparvovec. The data obtained in studies CT-AMT-010, without immunosuppression, and CT-AMT-011-01 and AMT-011-02, with immunosuppression, are considered comparable in terms of the decrease in TG levels (Figure 5). However, plasma TG levels subsequently increased in most patients around 12–14 weeks post-administration of alipogene tiparvovec. This was at a time interval when immune suppression had already been discontinued. This phenomenon was observed across the three studies and showed that fasting TG levels are not a sufficiently sensitive marker to monitor the long-term therapeutic effects of alipogene tiparvovec. Post-prandial chylomicron clearance kinetics has been recognized as the most relevant biological marker for the systemic activity of LPL during the clinical development of alipogene tiparvovec (50, 51). However, post-prandial chylomicron level measurements were included as endpoint only in the last of the three interventional studies, CT-AMT-011-02. The results have been reported (51) and show that the post-prandial chylomicron plasma levels are significantly reduced in all patients included in the study, independently of the presence of humoral (all patients) or cellular systemic (two on five patients) or local (three on five patients) immune responses against AAV1 (Figure 3), thus indicating that these responses had no influence on the efficacy of alipogene tiparvovec.

Furthermore, an ongoing study has shown a reduction in acute pancreatitis events in a series of more than 25 affected subjects (43). The analysis for a treatment-effect of Glybera taking into account exposure time demonstrated a significant and clinically relevant reduction in the risk of definite acute pancreatitis during various periods ranging from 2.5 to 10 years pre-treatment to post-treatment (median 2.9 years) (52).

## Effect of Immunosuppressants on Treatment-Emergent Immune Responses

Study CT-AMT-010, the first clinical study with AAV1–LPL^S447X^, was performed without treating the patients with immunosuppressants. In this study, no antibody or T-cells responses against LPL were found. However, antibodies against AAV1 capsid epitopes were observed in all patients whereas a T-cell response against AAV1 was detected only in four of the eight subjects.

As discussed, none of these immune responses raised specific safety concerns. However, they triggered more a concern about the efficacy of the product; especially the development of any AAV-specific T-cells (48) that were thought at the time to possibly hamper the expression of LPL. A similar T-cell response was observed in gene therapy trial for hemophilia B, in which an AAV2 vector was used to deliver the human coagulation factor IX (24, 41, 42). In this trial, two patients developed a T-cell response to AAV2 capsid proteins, which was not predicted from pre-clinical studies. In those two patients, transgene expression declined subsequently to pre-treatment levels. Based on these observations, it was concluded by the investigators that a cytotoxic T-lymphocyte response to the capsid may have contributed to a loss of transgene-expressing cells.

These discussions heavily influenced the decision to include an immunosuppressant regimen in the clinical study protocols for CT-AMT-011-01 as well as CT-AMT-011-02.

As a result of these discussions, 17 patients (12 in CT-AMT-011-01 and 5 in CT-AMT-011-02) treated with alipogene tiparvovec received a concomitant administration of immunosuppressants. Treatment-emergent anti-AAV1 antibody responses were observed in all the patients and were not affected by the immunosuppressants, neither during the time of administration nor after cessation of the administration. The AAV1–LPL^S447X^ vector used cannot induce expression of viral proteins in host cells. Hence, AAV1 capsid proteins are expected to be only transiently presented to the immune system of the recipient after injection of AAV1–LPL^S447X^ (3, 19, 53). Therefore, the immune suppressants drugs were given for a period of 12 weeks. A 12-week-period was considered to be sufficient for prevention of capsid immunogenicity, based on observations in monkeys (19) and the investigators early observations in humans that indicated TG levels started to rise after an initial decrease usually at some time between 4 and 12 weeks post-dosing. A combination of cyclosporine and MMF was chosen because this combination is widely used to prevent cytotoxic T-cell responses in transplant rejection. The doses of the immune suppressants proposed to co-administer with alipogene tiparvovec were according to approved doses for transplant rejection.

In study CT-AMT-011-01, humoral and cellular immune responses against AAV1 capsid proteins were observed. These responses were similar to those observed in study CT-AMT-010 although in study CT-AMT-011-01, a higher dose of AAV1–LPL^S447X^ was used (Figure 4). Therefore, the immunosuppressant regimen was further optimized in study CT-AMT-011-02 by starting administration of cyclosporine and MMF administration earlier before alipogene tiparvovec administration, and by adding a bolus injection of methylprednisolone at the time of alipogene tiparvovec administration. However, in study CT-AMT-011-02, comparable humoral and cellular immune responses against AAV1 capsid proteins were observed as in study CT-AMT-011-01 (Figure 4).

**Figure 6:**
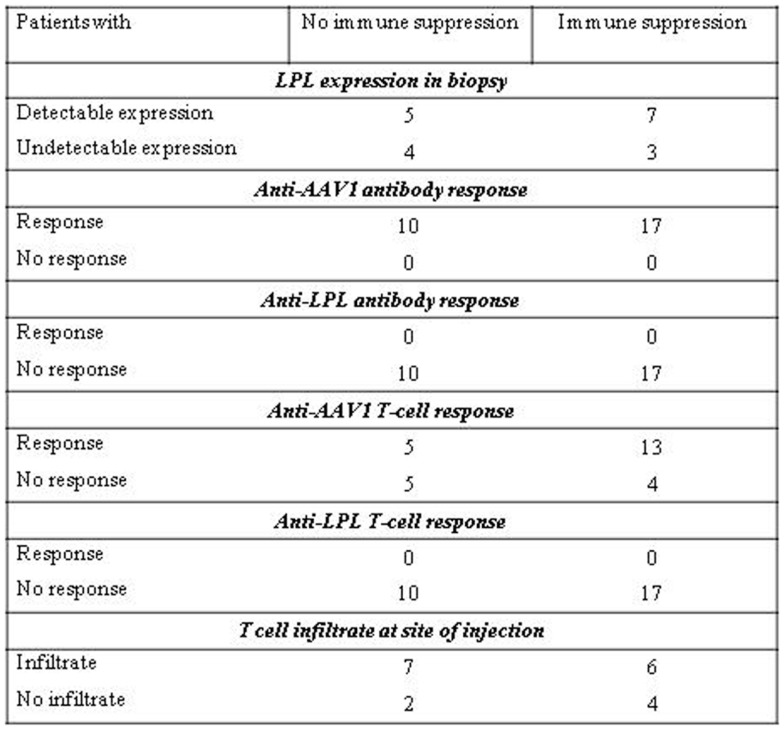
**Effect of immune suppression on LPL-expression and immune responses upon administration of alipogene tiparvovec**.

The effect of immunosuppressants on LPL-expression and immune responses upon administration of alipogene tiparvovec is summarized in Figure 6. In the clinical development of alipogene tiparvovec, no untoward side effects were observed that could be assigned to the use of prednisolone or one of the other immunosuppressants.

## Clinical Data

### Chemistry and hematological values

**Figure 7:**
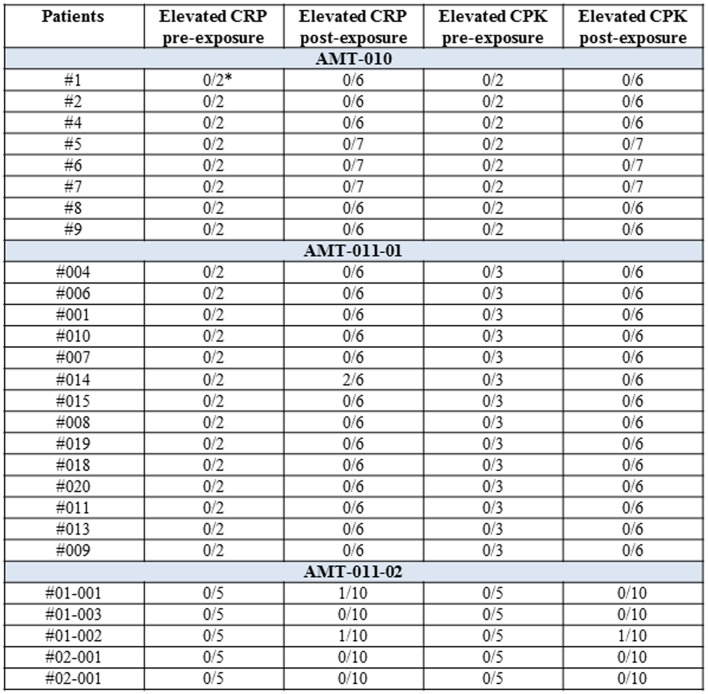
**A per-patient summary of CRP and CPK values pre- and post-exposure to alipogene tiparvovec**. Scheduled visits pre-treatment and post-treatment have been included. The post-treatment visits were at day 1 and weeks 1, 2, 4, 8, and 12 for studies CT-AMT-010 and CT-AMT-011-01. Weeks 14, 26, 39, and 52 were added for study CT-AMT-011-02. In the study AMT-011-01, the isolated elevation of CRP for subject 014 was concomitant with a transient medical condition. In the study AMT-011-02, the isolated elevation of CRP for subject 01-001 was concomitant with a transient medical condition. The isolated elevation of CRP and CPK for subject 01-002 was related to the general clinical condition of the subject.

Patients were monitored for up to 12 weeks post alipogene tiparvovec administration regarding routine hematology and biochemistry including CPK and CRP, increases of which are expected in case of local inflammatory damage at the injection site. In addition, other inflammatory markers such as neutrophil counts were also determined at several pre- and post-exposure occasions in the patients. None of the patients had neutrophil counts outside the normal range. A per-patient summary of the CRP and CPK data is given in Figure 7. The majority of the patients had normal CRP and CPK levels pre- and post-exposure to alipogene tiparvovec, suggesting that inflammatory reactions in the injected muscle were mild, if present, and of little clinical significance.

### Adverse events

Alipogene tiparvovec was administered via a one-time set of intra-muscular injections in the lower limbs. No consistent change in any laboratory parameter, linked to the administered product, was observed, including CPK. Injections were well tolerated with mild–moderate local injection site reactions lasting for a few days relating to the injection sites, and no change in muscle function. None of the patients showed clinical signs of persistent local inflammation at injection sites such as redness, swelling, warmth, pain, or dysfunction, upon administration of alipogene tiparvovec. There are to date no reports of muscle dysfunction in LPLD patients administered with alipogene tiparvovec.

The adverse reactions observed during the clinical development of alipogene tiparvovec are summarized in Figure S1 in Supplementary Material. Most of the adverse reactions were related to the administration procedure. All were of transient nature and resolved within days after the administration of the product. Only one serious adverse event involving muscle was seen, that was considered to be at least possibly related to alipogene tiparvovec by the Investigator (#01-002 in CT-AMT-011-02). In this subject, at 15 weeks post-administration, a transient rise in CPK, accompanied by a rise in CRP (Figure 7), was correlated with a low positive AAV cellular response associated with no LPL-related cellular or humoral significant response was seen in a complex of clinical symptoms and signs indicated as “polyarthralgia of imprecise origin.” The subject show sporadic systemic T-cell responses against AAV across the observation period at weeks 2, 8, 14, 39. Despite those T-cell responses, the muscle biopsy of this subject which was taken 30 weeks post alipogene tiparvovec showed robust LPL-expression. In addition, the subject did not show any anti-LPL cellular or humoral response (50). No adverse effect was observed that could have been related to the immune responses discussed in the previous paragraphs.

From the clinical data obtained across studies, we conclude that there is no clinical untoward impact of the delivery of alipogene tiparvovec.

## Discussion

To evaluate the immunogenicity of alipogene tiparvovec, an extensive testing program was performed that included the analyses of antibody and T-cell responses to LPL as well as to AAV1. Antibody and cellular responses were measured pre-exposure to alipogene tiparvovec, and at various occasions post-exposure. In addition, biopsies were taken from the injected muscle and non-injected muscle as control, to evaluate local immune and inflammatory processes. Finally, CPK and CRP levels and neutrophil counts were measured, and patients were clinically evaluated for local and systemic symptoms indicative for inflammatory or immune reactions. Expression of LPL in the injected muscle as well as the improvement of post-prandial chylomicrons clearance in plasma was used as biochemical markers for efficacy.

The success of *in vivo* gene therapy not only depends on the ability to control the immune response toward the vector, but also to monitor any potential reaction to the therapeutic protein expressed from the transgene. The data obtained from patients with LPL-deficiency who received a single treatment with multiple injections of alipogene tiparvovec, support the initial expectation that the protein product is minimally immunogenic, if at all: neither treatment-emergent antibody responses against LPL nor T-cells responses against LPL were found. Thus, the expressed protein itself appears to be non-immunogenic.

Concerning anti-AAV immunity, the majority of healthy individuals are exposed to AAV during lifetime (2, 54). Hence it is not surprising that 15 of 27 patients had pre-existing antibodies against AAV1. There was no difference among patients with or without these antibodies with regard to detectable LPL-expression in the biopsies or improvement of post-prandial chylomicron clearance, suggesting that pre-existing anti-AAV1 antibodies did not appear to prevent LPL transgene expression and clinical efficacy. Therefore, pre-existing anti-AAV1 antibodies likely have no effect on the efficacy of alipogene tiparvovec following intra-muscular administration. All patients showed treatment-emergent antibody responses to AAV1 and there was no difference in anti-AAV1 antibody response between the various dosing cohorts.

The markers that were used to demonstrate LPL-expression and functionality after the delivery of alipogene tiparvovec are complex. Therefore, the present report is focusing on LPL-expression in muscle biopsies and post-prandial chylomicron clearance as marker for systemic LPL activity. For the direct measurement of LPL-expression, we made use of biopsies that were obtained at different time points up to 12 months after drug administration. However, and as described above, not all patients gave their consent to this procedure which can present some variability in the execution. Nineteen patients provided their consent and in total 20 biopsies were obtained, which all were examined for LPL-expression. In 12 of the 20 biopsies, LPL-expression was found irrespective of the presence of antibodies against AAV1. The second biochemical marker for successful gene delivery in this review is the level of clearance of post-prandial chylomicrons in plasma, which reflect the systemic activity of LPL (49). This marker was however, included only in the study CT-AMT-011-02. The post-prandial chylomicrons plasma levels were shown to be significantly reduced in all five patients included in this study, independently of the presence of anti-AAV1 antibodies.

Altogether, these observations demonstrate that anti-AAV1 antibody responses did not exclude sustained transgene expression nor did impair the systemic biological activity of the expressed protein. Our results further support that treatment-emergent anti-AAV1 antibody responses do not necessarily have any influence on the long-term efficacy and safety of AAV-based gene therapy.

Treatment-emergent T-cell responses against AAV1 capsids were measured with the ELISpot assay and were observed in 18 of the 27 patients. However, it has to be noted that the patients treated with the higher dose, 1 × 10^12^ gc/kg, were more prompt to develop an AAV1-specific T-cells responses. Somewhat higher T-cell responses were noticed in some patients upon cessation of the immunosuppressants, pointing to a suppressive effect on the T-cell responses. It is therefore not possible to make definitive conclusions regarding the effect of immunosuppressants on T-cell responses to AAV1. The immunosuppressants did not affect antibody levels as was expected since the regimen was mainly aiming at reducing potential T-cell responses. MMF and cyclosporine are both described to be effective in suppressing cytotoxic T-cell responses. MMF has an effect on B-cell proliferation, because it inhibits *de novo* guanosine nucleotide synthesis, a pathway commonly required for both T- and B-cell proliferation. Cyclosporine specifically inhibits T-cell activation by inhibiting IL-2 production and exerts limited, if any, effect on B-cell proliferation. Thus, the increased anti-AAV1 titers are not surprising. Similar observations were made in a study in monkeys (52) when administrating AAV8-hFIX together with immunosuppressants consisting of MMF and tacrolimus. In the third study of the three interventional studies for alipogene tiparvovec, CT-AMT-011-02, prednisolone was administered in the form of a bolus injection to prevent the release of substances that mediate inflammation and to enhance the potency of the other immunosuppressants used.

Our studies should be considered in the context of the growing body of clinical and pre-clinical studies evaluating the role of capsid-specific T-cells in AAV gene. The immunogenicity data found in the clinical studies conducted with AAV-based vectors in human, show that immune responses against AAV capsid proteins can vary widely and amongst others are influenced by the target organ, route of delivery, and dosing schedule. When targeting the muscle in humans, T-cell responses directed to the capsid antigen were documented in AAT-deficient subjects receiving intra-muscular injection of an AAV1–AAT vector (25, 55), in study on AAV1-α-sarcoglycan in limb-girdle muscular dystrophy subjects (35) and in our own LPL-deficient subjects. The controversial aspect of the capsid-specific T-cell hypothesis is whether the vector sensitizes transduced cells to become targets for CTL-mediated clearance by virtue of MHC presentation of peptides from the input capsid protein. Also, immune modulation was used which could have impacted on the AAV-specific immune responses, our study provides the most direct and extensive test of this hypothesis because we observed transgene expression until 52 weeks (long-term follow-up) after injection of AAV1–LPL^S447X^ in the muscle, despite the detection of circulating T-cell specific for AAV capsid peptides in some subjects and persistent focal infiltrates in all subjects for whom transgene expression was detected. These data clearly demonstrate that transgene expression can persist, despite the presence of capsid-specific T-cells and cellular infiltrates. Sustained transgene expression in the presence of T-lymphocyte responses have been reported in the literature in experimental animals and in different tissues (56, 57) and humans (25, 35, 37). However, whereas attention has been focused initially on the AAV capsid as target of an undesirable T-cell response (24, 41, 42), observations made by the groups of Dr C. Walker (58) and supported by observations of others (59) suggest that loss of function and programed death by most tissue-infiltrating AAV-primed T-cells seem to argue against their direct participation in clearance of AAV-vector-transduced target cells. It has been described that T-cells in AAV-vectors related infiltrates present characteristics of anergy (55). Such T-cell infiltrates are therefore generally considered as unable to initiate cellular self-destruction and therefore do not impact on efficacy of transgene expression.

Another mechanism via which T-cells may affect LPL-expression is stimulating the proliferation and differentiation of B cells that subsequently form antibodies against AAV1. However, as is discussed above, anti-AAV1 antibodies seem to have no impact on LPL-expression and there is no evidence whether antibody-dependent cell-mediated cytotoxicity played a role. As mentioned before, a sustained long-term transgene expression was observed after intra-muscular injection, despite the presence of circulating antibodies directed against the AAV1 capsid peptides. The results obtained with alipogene tiparvovec demonstrate that the presence of the anti-AAV1 humoral immune response had no apparent influence on the long-term efficacy of the therapy. The same observation has been reported in other clinical studies (25, 35, 37, 47, 55).

Multiple intra-muscular injections of the vector and supposed inflammatory and immune reactions ensuing at the injection sites raise concerns about inflammatory damage in the injected muscle. However, except for transient mild local procedural symptoms at the injection sites, no clinical symptoms such as swelling, pain, or dysfunction pointing at inflammatory damage were observed in the patients. In addition, serial monitoring of CRP and CPK revealed normal levels of these markers in most patients. Occasional elevations of CRP and CPK were seen in two and in one patient, respectively, without any clinical correlation. In addition, though a mild mononuclear infiltrate was observed in 14 of the 19 patients of whom a biopsy was obtained, this infiltrate lacked substantial cytotoxic T-cells activity. Hence, no clinical, biochemical, histochemical, or immunological evidence for inflammatory muscle damage at the injections sites was found.

All together, the data collected on systemic and local immune responses induced by intra-muscular injection of alipogene tiparvovec demonstrate the absence of impact on safety and did not compromise LPL transgene expression. These findings indicate that muscle-directed AAV-based gene therapy through the intra-vascular route remains a promising approach for the treatment of human diseases.

## Regulatory Perspective

During the assessment of the Glybera marketing authorization dossier, the fact that no responses had been seen against the expressed LPL protein was considered as a positive safety asset and no material concerns were expressed.

The necessity of using immunosuppressants was not proven during the clinical development of Glybera since the regimen seemed not to improve efficacy whilst having a major negative impact on the safety aspects. It remains questionable whether it makes clinical sense to co-administer immunosuppressants with any AAV-based vector. However, in the case of Glybera, since LPLD is such a rare disease, it will not be possible to further assess long-term clinical efficacy in absence of any immunosuppressants within the present indication.

During the regulatory review by the European Medicines Agency, multiple questions arose on whether the cellular responses to the viral capsid proteins could have any meaningful negative effect on the long-term safety or efficacy of alipogene tiparvovec. Our data clearly demonstrate that transgene expression can persist, despite the presence of capsid-specific T-cells and cellular infiltrates and without apparent toxicity or attenuation of transgene expression. Furthermore, the purity of the vector preparations (in terms of total amount of viral proteins injected versus dose in genome copies) and impurities profile of the vectors used in the various clinical trials described in the literature may be very divergent and therefore may lead to very different immune responses. Nonetheless, the scientific debate has been powerfully influenced by previous findings with other vectors and by the hypothesis that the vector sensitizes transduced cells to become targets for CTL-mediated clearance. Therefore, since the safety data on alipogene tiparvovec have been collected in a small number of patients, their clinical relevance and possible interpretations were considered not fully unequivocal and further data collection has been requested by the European Medicine Agency post-approval of Glybera^®^. Such data will be collected from all treated patients in future in a LPLD registry, thus allowing for long-term data analysis.

## Conflict of Interest Statement

The funding body (uniQure) was involved in all aspects of the study. The authors (Valerie Ferreira, Florence Salmon, and Harald Petry) are employes of uniQure.

## Supplementary Material

The Supplementary Material for this article can be found online at http://www.frontiersin.org/Journal/10.3389/fimmu.2014.00082/abstract
