# Supplementary figures and images for: Immune Responses to AAV-Vectors, the Glybera Example from Bench to Bedside
Source: Front Immunol. 2014 Mar 3;5:82. doi: 10.3389/fimmu.2014.00082 (PMC3939780; doi:10.3389/fimmu.2014.00082)

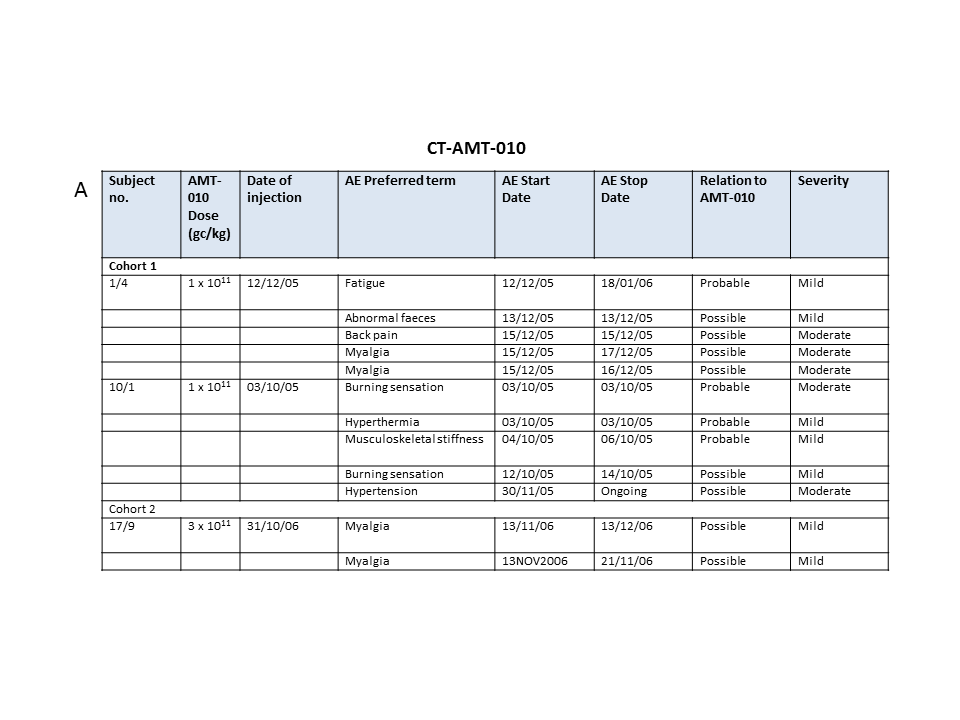

Supplement: Supplementary file 1 [file Presentation_1.ZIP › Figure S1-A.TIF]

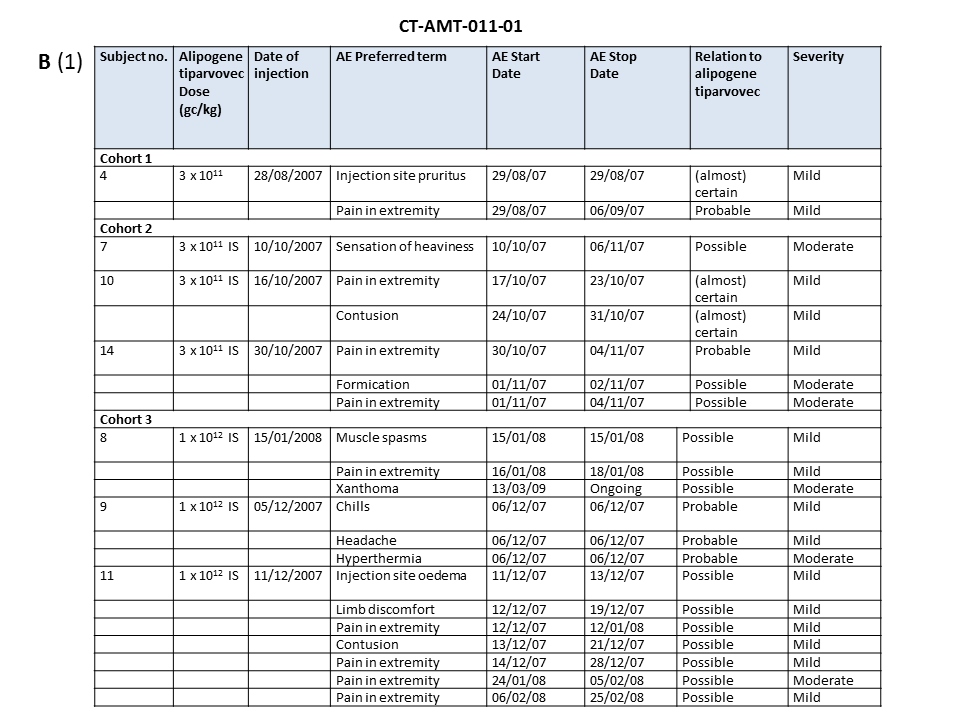

Supplement: Supplementary file 1 [file Presentation_1.ZIP › Figure S1-B(1).TIF]

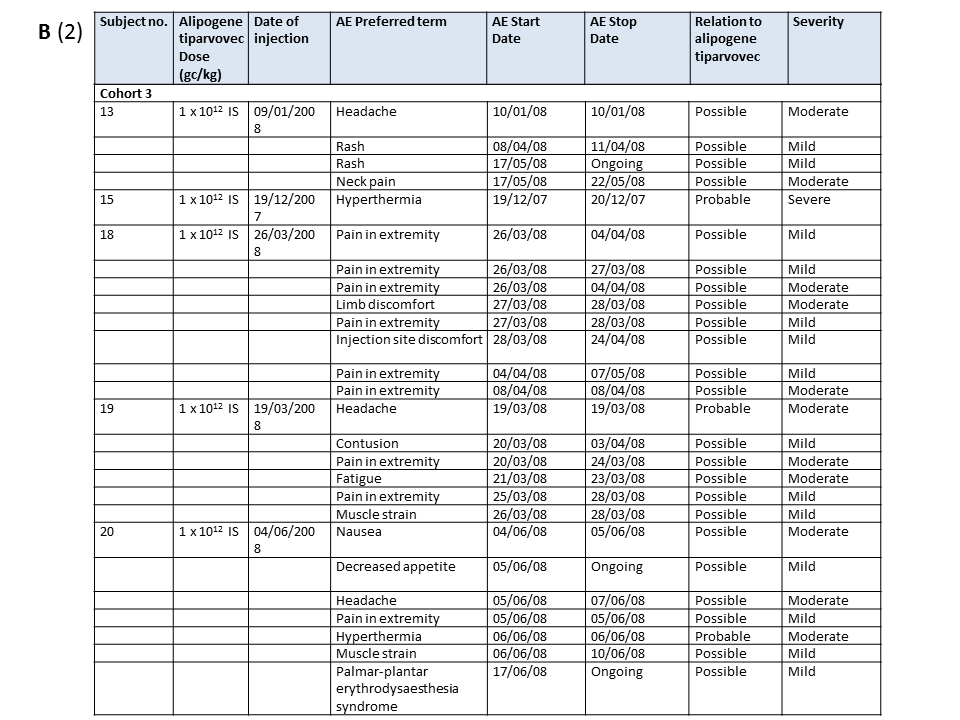

Supplement: Supplementary file 1 [file Presentation_1.ZIP › Figure S1-B(2).TIF]

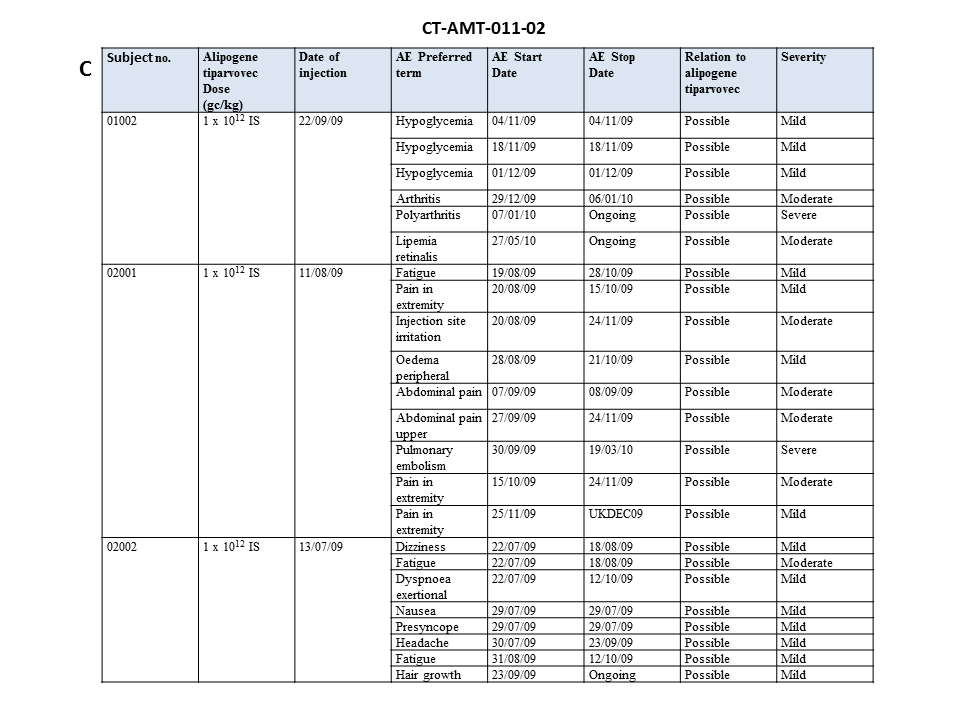

Supplement: Supplementary file 1 [file Presentation_1.ZIP › Figure S1-C.TIF]
